# On fast simulation of dynamical system with neural vector enhanced numerical solver

**DOI:** 10.1038/s41598-023-42194-y

**Published:** 2023-09-14

**Authors:** Zhongzhan Huang, Senwei Liang, Hong Zhang, Haizhao Yang, Liang Lin

**Affiliations:** 1 School of Computer Science and Engineering, Sun Yat-sen University, Guangzhou, China; 2 Department of Mathematics, Purdue University, West Lafayette, IN USA; 3 Mathematics and Computer Science Division, Argonne National Laboratory, Lemont, IL USA; 4 Department of Mathematics, University of Maryland College Park, College Park, MD USA

**Keywords:** Mathematics and computing, Applied mathematics, Computational science, Computer science

## Abstract

The large-scale simulation of dynamical systems is critical in numerous scientific and engineering disciplines. However, traditional numerical solvers are limited by the choice of step sizes when estimating integration, resulting in a trade-off between accuracy and computational efficiency. To address this challenge, we introduce a deep learning-based corrector called Neural Vector (NeurVec), which can compensate for integration errors and enable larger time step sizes in simulations. Our extensive experiments on a variety of complex dynamical system benchmarks demonstrate that NeurVec exhibits remarkable generalization capability on a continuous phase space, even when trained using limited and discrete data. NeurVec significantly accelerates traditional solvers, achieving speeds tens to hundreds of times faster while maintaining high levels of accuracy and stability. Moreover, NeurVec’s simple-yet-effective design, combined with its ease of implementation, has the potential to establish a new paradigm for fast-solving differential equations based on deep learning.

## Introduction

**Figure 1:**
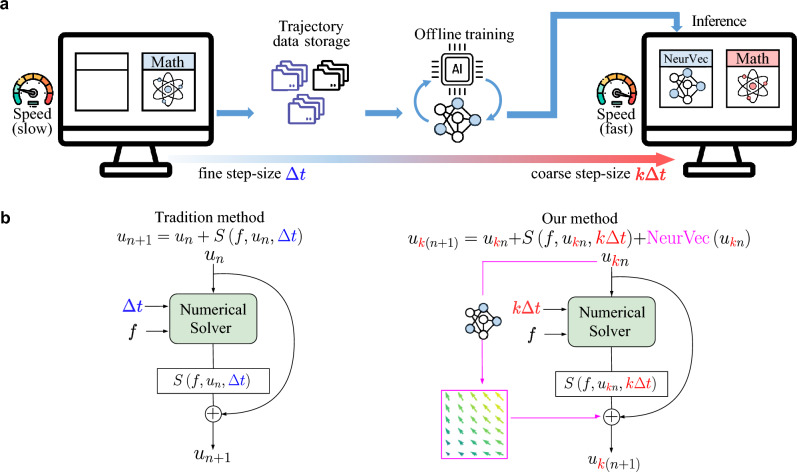
The structure of NeurVec. (**a**) Deployment of NeurVec for fixed step size solver. During offline training, NeurVec learns from the solutions of high accuracy to characterize the error caused by the use of a coarse step size. During inference, NeurVec applies to the solver and integrates with the coarse step size. (**b**) Comparison of the tradition solver and solver armed by NeurVec. Left: Forward loop of traditional solver with step size $\Delta t$. Right: Forward loop of NeurVec with step size $k\Delta t$.

Dynamical systems are widely used to characterize the time dependence of the physical states and to model phenomena that change with time^1–3^. Studying the temporal evolution of dynamical systems and their statistics can help uncover the physics behind the dynamics and predict future states of the systems^3^. Typically, a time-dependent *d*-dimensional state *u*(*t*) is depicted by a system of ordinary differential equations (ODEs):

1 $$\begin{aligned} \frac{\text {d}\textbf{u}(t)}{\text {d}t} = \textbf{f}[\textbf{u}(t)], \quad \textbf{u}(0) = \textbf{c}_0, \end{aligned}$$

where $\textbf{c}_0$ represents an initial condition. This system arises in many science and engineering fields such as climate change^4,5^, air pollution,^6,7^stable financial systems^8^, power grid management^9,10^, transportation networks^11,12^, and medical analysis and drug discovery^13–15^. To obtain a numerical solution of (1), one may employ an integration method^16,17^ (Fig. 1b) given by the iterative formula

2 $$\begin{aligned} \textbf{u}_{n+1} = \textbf{u}_{n} + S(\textbf{f}, \textbf{u}_{n}, \Delta t_n), \quad \textbf{u}_0 = \textbf{c}_0, \quad n=0,1,\cdots , \end{aligned}$$

where *S* represents a numerical scheme (for example, $S(f, \textbf{u}_{n}, \Delta t_n):=f(\textbf{u}_{n})\Delta t_n$ when the Euler method^18^ is used), $\Delta t_n$ is the step size of the *n*th time step, and $\textbf{u}_{n}\in {\mathbb {R}}^d$ is an approximated solution at time $\sum _{i=0}^n \Delta t_n$. When approximating a solution at a specific time given an initial condition, we readily customize accuracy and speed via tuning integration strategies (e.g., different scheme *S* and step size $\Delta t_n$ selection). However, many real-world applications^19–22^ require simulating many trajectories. In particular, large-scale simulation, which produces forecasts on a set of initial conditions simultaneously, is more useful for these applications. Compared with a single simulation, ensemble-based large-scale simulation is a computationally challenging problem but plays a critical role in a variety of demanding applications. For illustration, we present a few scenarios of such simulation.

### Fast simulation

Sine late 2019, the epidemic of COVID-19 has raged around the world, hitting the global health and economy^23^. Scientists need to perform simulations of virus propagation under thousands of different circumstances^24,25^. These predictions provide the scientific reference for governments to make quick responses and control policies^26,27^. The virus, such as the Delta and Omicron variants, spreads rapidly and mutates frequently^28^. A slow simulation may lead to a delay in decision-making and worsen the situation.

### Synchronous simulation

 Particle systems are a graphical technique that simulates complex physical effects (such as smoke^29^, water flow^30^, and object collision^31^). This is widely used in applications in games, movies, and animation^32^. These applications involve synchronously simulating thousands of particles at one time. Yet supporting the real-time simulation of these particle motions with satisfactory visual enjoyment is expensive.

### Reliable model

 Weather forecasting is beneficial for making a proper plan for production and living^33,34^. A single forecast of the weather model essentially suffers from considerable errors introduced by the highly simplified model formulation and the chaotic nature of the atmosphere evolution equations. In order to avoid a misleading single forecast, ensemble forecasting^35–37^ presents a range of possible future weather states through conducting simulations from multiple initial conditions and models.

To meet the demands of these applications, we need to develop a fast solver that is capable of simultaneously simulating the dynamical system over a large batch of initialization data. The advances in processors, such as graphics processing units (GPUs)^38^, tensor processing units^39^, and natural graphics processing units^40^, provide the possibility of accelerating the numerical computation via parallel computing of batch data. However, most hardware implements restrictive SIMD-based (single instruction, multiple data) models^41^. The numerical method that needs individual processing of each trajectory is not appropriate for SIMD processors directly. For example, the adaptive time-step integrator (e.g., the Runge–Kutta–Fehlberg method^42^) determines a step size at each step based on an estimate of the local error, making the iterative computation in Eq. (2) asynchronous for each trajectory in the batch and affecting the efficiency of parallel computing. On the other hand, we may control the step size to be the same at each step for all trajectories by adding one dimension to Eq. (2), for example, $\textbf{u}_{n+1}\in {\mathbb {R}}^{N\times d}$ with *N* representing the batch size. Controlling the step size requires considering a combined ODE system and estimating the error on all batch elements^43^. The step size is limited by the largest local truncation error in a batch, making it difficult to use a large step size especially when the batch size is large^43,44^. If the step size is always small in each step, it becomes slow for integration. Therefore, SIMD processors prefer a fixed time-step integrator (i.e., $\Delta t:=\Delta t_1=\Delta t_2=\cdots$),

3 $$\begin{aligned} \textbf{u}_{n+1} = \textbf{u}_{n} + S(\textbf{f}, \textbf{u}_{n}, \Delta t), \quad \textbf{u}_0 = \textbf{c}_0, \quad n=0,1,\cdots . \end{aligned}$$

However, a fixed step size integrator encounters a trade-off^44,45^ on step size between accuracy and computational efficiency: a large step size has a fast simulation but leads to a less accurate solution, while a small step size has a slow simulation but achieves a more accurate solution (see Table 1 for the comparison of evaluation time and theoretical error between the traditional solvers with fine or coarse step size). This trade-off limits the feasibility of large-scale simulation if high accuracy is required.

To break through this speed-accuracy trade-off, in this paper we propose an open-source and data-driven corrector, called neural vector (NeurVec), which enables integration with coarse step size while maintaining the accuracy of fine step size in large-scale simulations. We emperically demonstrate that NeurVec is capable of overcoming the stability restriction of explicit integration methods for ODEs. The deployment of NeurVec comprises offline training and inference (Fig. 1a). During offline training, NeurVec is trained with the accurate solution, while during inference NeurVec is employed to the solver to compensate for the error caused by the coarse step size. Our results on some complex dynamical system benchmarks show that NeurVec is capable of learning the error term and accelerating the large-scale simulation of dynamical system significantly. Additionally, we have found that NeurVec not only overcomes the stability restriction of explicit integration methods for ODEs but also exhibits excellent generalization capabilities.

Some related works^46–48^ centered around purely data-driven methods for enhancing the accuracy of learning unknown dynamics from data. These approaches employ neural networks to characterize the system dynamics. For example, Ref.^46^ improve long-term predictive modelling of nonlinear systems approximated by neural networks, by introducing supplementary regularization loss terms. In Ref.^48^, neural networks are fused with existing coarse modeling techniques to improve the accuracy of a reduced/coarse model. Different from the previous works that focus on enhancing the accuracy of learning unkown dynamics^49^, our objective is to accelerate the numerical simulation of multiple trajectories, each initialized differently, for dynamics that are already known. In our approach, we use neural networks to compensate the time integration errors that arise because of large time step sizes. There are some previous works also consider accelerating the solution estimation via deep learning, but they emphasize adopting pure data-driven approaches^3,45,50^, without using any explicit formula of the equation. Because of chaos^51–53^ (solution is sensitive to small perturbations) and stiffness^44,54^ (solution is unstable unless a sufficiently small step size is used), the pure data-driven method still suffers from large errors in prediction, especially for long-term prediction^55^.

## Framework of NeurVec

In this study, we aim to discretize and solve the differential equation given by Eq. (1) using a larger step size, specifically *k* times the step size ($k\Delta t$). To illustrate this approach, we consider the Euler method as an example, and use Taylor expansion to obtain:

4 $$\begin{aligned} \textbf{u}(t+k\Delta t) = \underbrace{\textbf{u}(t) + \textbf{f}[\textbf{u}(t)]\cdot k\Delta t}_{\text {For Euler method}} + \sum _{n=2}^\infty \underbrace{ \frac{1}{n!} \frac{\text {d}^n}{\text {d}t^n}\textbf{u}(t)\cdot [k\Delta t]^n}_{\text {Error term err}_n }. \end{aligned}$$

By neglecting all high-order error terms $\text {err}_n$ and discretizing $\textbf{u}$, we can obtain the corresponding Euler method. However, when using a large step size of $k\Delta t$, discarding all high-order error terms will significantly reduce the accuracy of the Euler method, leading to poor predictions. Notably, from Eq. (1), we have $\text {err}_n (k,\Delta t, \textbf{u}(t)) \triangleq \frac{1}{n!} \frac{\text {d}^n}{\text {d}t^n}\textbf{u}(t)\cdot [k\Delta t]^n = \frac{1}{n!} \frac{\text {d}^{n-1}}{\text {d}t^{n-1}}\textbf{f}[\textbf{u}(t)]\cdot [k\Delta t]^n$, which depends on $\textbf{u}(t)$ and the constants *k* and $\Delta t$. Therefore, we consider utilizing a corrector called NeurVec by a neural network with input $\textbf{u}(t)$ to approximate $\sum _{n=2}^\infty \text {err}_n$. In other words, the corrector NeurVec, in the form of a vector function, is directly added to the estimated solution to compensate for the error caused by the use of the coarse step size (Fig. 1b). Following this idea, other forward numerical solvers besides the Euler method can also benefit from NeurVec to compensate for errors. Specifically, NeurVec, a neural network parameterized by $\Theta$, maps from the state ${\mathbb {R}}^d$ to the error correction ${\mathbb {R}}^d$ (see the Methods section) and is added to the iterative formula of the solver with the step size $k\Delta t$ to get the solution $\{\hat{\textbf{u}}_{kn}\}_{k=0}^\infty$, i.e.,

5 $$\begin{aligned} \hat{\textbf{u}}_{k(n+1)} = \hat{\textbf{u}}_{kn} + S(\textbf{f}, \hat{\textbf{u}}_{kn}, k\Delta t) + \text {NeurVec}(\hat{\textbf{u}}_{kn};\Theta ), \quad \hat{\textbf{u}}_0 = \textbf{c}_0, \quad n=0,1,\cdots . \end{aligned}$$

With NeurVec, we just need to estimate the solution on every *k* steps instead of step by step as in Eq. (3). NeurVec is trained from the more accurate solutions with fine step size $\Delta t$ to characterize the error caused by the use of the coarse step size $k\Delta t$. The parameter $\Theta$ in NeurVec can be optimized by minimizing the mean squared difference between the predicted error and the error of the solver with the coarse step size:

6 $$\begin{aligned} \min _{\Theta }\frac{1}{G}\sum _{n=1}^G\big \Vert \text {NeurVec}(\textbf{u}_{kn};\Theta )-\big (\textbf{u}_{k(n+1)}-\textbf{u}_{kn} - S(\textbf{f}, \textbf{u}_{kn}, k\Delta t)\big )\big \Vert _2^2, \end{aligned}$$

where *G* is the number of training samples $\{\textbf{u}_s\}_{s=1}^{(G+1)k}$ from the fine step size. Table 1 displays a comparison of evaluation time and theoretical error between the traditional solver and NeurVec. We use $\epsilon$ to denote the runtime ratio of NeurVec to the scheme *S*. NeurVec inevitably increases the relative time complexity for each step by $\epsilon$ since an additional computation module is used (see supplementary material for details). When $k> (1+\epsilon )$, NeurVec with the coarse step size $k\Delta t$ is faster than the solver with the fine step size $\Delta t$, while achieving comparable accuracy. Moreover, the runtime increment $\epsilon$ of NeurVec can be lessened. For example, the more complicated scheme *S* increases the time complexity, and built-in parallel computing in Pytorch^56^, TensorFlow^57^, or other deep learning frameworks enables smaller time complexity of NeurVec. As we will see in the Results section, $\epsilon < 1$ uniformly, so NeurVec can accelerate the solver when $k\ge 2$. To characterize the solution error of NeurVec, we consider the Euler method, a simple ODE solver, as a proof of concept. The global truncation error of the Euler method linearly grows with the step size^16^, namely, ${\mathcal {O}}(\Delta t)$ when the step size is $\Delta t$ and ${\mathcal {O}}(k\Delta t)$ when the step size is $k\Delta t$. In our theory, we show that NeurVec of sufficient width can achieve an error of ${\mathcal {O}}(\Delta t)$ when the step size is $k\Delta t$, which breaks the accuracy-speed trade-off.

**Table 1:** Comparison of evaluation time and theoretical error (based on the Euler scheme) among the numerical solvers with fine $(\Delta t)$ or coarse step size $(k\Delta t)$ and NeurVec $(k\Delta t)$. Here $\epsilon$ denotes the ratio of the runtime of NeurVec to that of scheme *S* for one step. The fixed step size solvers suffer from the accuracy-speed trade-off on the step size. NeurVec learns from the solutions of fine step size. Then NeurVec is applied to the solver and integrates with the coarse step size ($k\Delta t$) but still has the theoretical accuracy of the fine step size, ${\mathcal {O}}(\Delta t)$.

Method	Step size	Evaluation time	Theory error (Euler scheme)
Fixed step size solver (fine step size)	$\Delta t$	${\mathcal {O}}(1/\Delta t)$	${\mathcal {O}}(\Delta t)$
Fixed step size solver (coarse step size)	$k\Delta t$	${\mathcal {O}}(1/(k\Delta t))$	${\mathcal {O}}(k\Delta t)$
NeurVec (coarse step size)	$k\Delta t$	${\mathcal {O}}((1+\epsilon )/(k\Delta t))$	${\mathcal {O}}(\Delta t)$

## Results

We verify the capabilities of NeurVec in two aspects: (1) NeurVec is capable of stabilizing and accelerating the simulation on widely used numerical solvers with consistent performance improvement; and (2) NeurVec can be applied effectively to various complex dynamical system benchmarks.

To illustrate the performance of NeurVec, we employ a simple network structure, a one-hidden-layer fully connected neural network^44^, to model NeurVec, where the number of the hidden neurons is 1,024 and a rational function^58^ is used (see the Methods section for details). The training and inference of NeurVec are all performed on a single GeForce RTX 3080 GPU with a memory of 10 gigabytes. The simulations in the training and testing sets are uniformly sampled every time interval $\eta$, and hence we have discrete solutions on the time $0, \eta , 2\eta , \cdots$. To compare the speed, we average the clock time of simulations over 70 trials. The complete statistical tests can be found in the supplementary material.

In the preceding sections we used $\Delta ^{\textrm{F}}t$ and $\Delta ^{\textrm{C}}t$ to distinguish the fine step size from the coarse one used in traditional solver (e.g., $\Delta ^{\textrm{F}}t=\Delta t$ and $\Delta ^{\textrm{C}}t=k\Delta t$ in our previous notation). $\Delta ^{\textrm{NV}}t$ represents using NeurVec and integrating with step size $\Delta ^{\textrm{NV}}t$.

**Figure 2:**
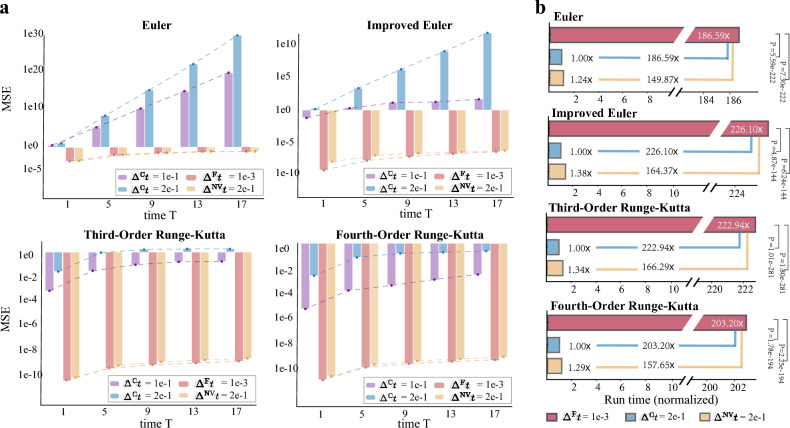
Application of NeurVec on different numerical solvers. (**a**) The mean square error (MSE) between the reference solution and the numerical solutions with different configurations (step size $\Delta ^{\textrm{C}}t = 1e-1$, $\Delta ^{\textrm{C}}t=2e-1$, $\Delta ^{\textrm{F}}t=1e-3$, and NeurVec ($\Delta ^{\textrm{NV}}t = 2e-1$)) on the spring-chain system, averaged over 10.5k simulations. The reference solution is obtained by using the 4th-order Runge–Kutta with step size $1e-4$. NeurVec is trained on the simulations of $\Delta ^{\textrm{F}}t=1e-3$. The numerical solution of $\Delta ^{\textrm{C}}t=2e-1$ becomes unstable using Euler or the improved Euler formula, while NeurVec ($\Delta ^{\textrm{NV}}t=2e-1$) achieves a stable solution with accuracy comparable to that of $\Delta ^{\textrm{F}}t=1e-3$. (**b**) The (normalized) runtime of the numerical solver with $\Delta ^{\textrm{F}}t=1e-3$, $\Delta ^{\textrm{C}}t=2e-1$, and NeurVec ($\Delta ^{\textrm{NV}}t=2e-1$). The runtime of $\Delta ^{\textrm{C}}t=2e-1$ is benchmarked to one unit. NeurVec ($\Delta ^{\textrm{NV}}t=2e-1$) has accuracy similar to that of $\Delta ^{\textrm{F}}t=1e-3$ and is over 150 times faster.

### NeurVec for different numerical solvers

We demonstrate the performance of NeurVec on widely used numerical solvers with consistent performance improvement. We perform NeurVec with four solvers: Euler, improved Euler, third-order Runge–Kutta (RK3), and fourth-order Runge–Kutta (RK4) (see the Methods section) on a high-dimensional spring-chain system^59^. The spring-chain system we consider describes the motion of *d* masses linked by $d+1$ springs, and the springs are placed horizontally with the two ends connected to a fixed wall. The ODE of the system is given by

7 $$\begin{aligned} \frac{\text {d} q_i}{\text {d}t} = \frac{p_i}{m_i}, \quad \frac{\text {d} p_i}{\text {d}t} = k_i(q_{i-1}-q_i)+k_{i+1}(q_{i+1}-q_i), \quad i=1,2,\cdots , d, \quad q_0=q_{d+1}=0, \end{aligned}$$

where the variables $q_i$ and $p_i$ represent position and momentum of the *i*th mass, respectively, $i=1,\ldots , d$. Here $m_i$ and $k_i$ are the mass of the *i*th mass and force coefficient of the *i*th spring, respectively, and they are randomly and uniformly sampled (for exact value, see the supplementary material). We first introduce the training dataset to train NeurVec and the testing dataset for evaluation. The training and testing simulations are uniformly sampled every time interval $\eta = 2e-1$. The initial states are sampled randomly from uniform distribution $\pi :={\mathcal {U}}([-2.5, 2.5]^d\times [-2.5, 2.5]^d)$. We set the dimension $d=20$ so the dimension of the state is 40. Given a scheme *S*, the training dataset is generated by *S* with $\Delta ^{\textrm{F}}t = 1e-3$. The reference simulations in the testing set are generated by RK4 with sufficiently small step size $1e-4$ (see the supplementary material).

Next, we demonstrate the performance of NeurVec in terms of accuracy and speed. NeurVec learns from the simulations of $\Delta ^{\textrm{F}}t=1e-3$ and is applied to the numerical solver with $\Delta ^{\textrm{C}}t=2e-1$. We characterize accuracy by the MSE between the reference and the simulated solution. In the short-term simulation on the time interval [0, 17], the numerical solutions of the coarse step size ($\Delta ^{\textrm{C}}t \ge 1e-1$) incur considerable simulation error and become unstable if Euler and the improved Euler are used (Fig. 2a). By contrast, NeurVec ($\Delta ^{\textrm{NV}}t=2e-1$) achieves a stable solution with accuracy comparable to that of the fine step size $\Delta ^{\textrm{F}}t=1e-3$ (Fig. 2a), which means that NeurVec can overcome the stability restriction. These observations indicate that NeurVec learns the error distribution from the fine step size dataset and compensates for errors caused by the use of the coarse step size, demonstrating that NeurVec is compatible to these solvers. To better display the runtime, we benchmark the runtime of $\Delta ^{\textrm{C}}t=2e-1$ as one unit. The use of NeurVec increases for a certain runtime ($\epsilon \le 0.38$) for a single step (compared with $\Delta ^{\textrm{C}}t=2e-1$), but NuerVec has accuracy comparable to that of $\Delta ^{\textrm{F}}t=1e-3$, which needs 200 steps for integrating over the time interval $2e-1$. The runtime of $\Delta ^{\textrm{F}}t=1e-3$ is much higher than that of NeurVec ($\Delta ^{\textrm{C}}t=2e-1$) (*P* value $\ll$ 0.001 under two-sided t-tests), and NeurVec enables these numerical methods to have more than 150$\times$ speedup on the spring-chain systems (Fig. 2b).

### NeurVec on complex dynamical systems

We verify the effectiveness of NeurVec on challenging complex systems, including the classical chaotic system Hénon–Heiles system, elastic pendulum, and *K*-link pendulum. For more challenging systems, such as the Kuramoto-Sivashinsky equation (KSE), please refer to the supplementary materials. The chaotic system is sensitive to perturbation to the initial state, and small errors are increased exponentially by the dynamics. For all of these examples, we generate the testing set using RK4 with step size $1e-4$ while the training set is generated with $\Delta ^{\textrm{F}}t=1e-3$. The initial conditions are randomly and uniformly sampled on a range of values (see the supplementary material). NeurVec is applied to RK4.

**Figure 3:**
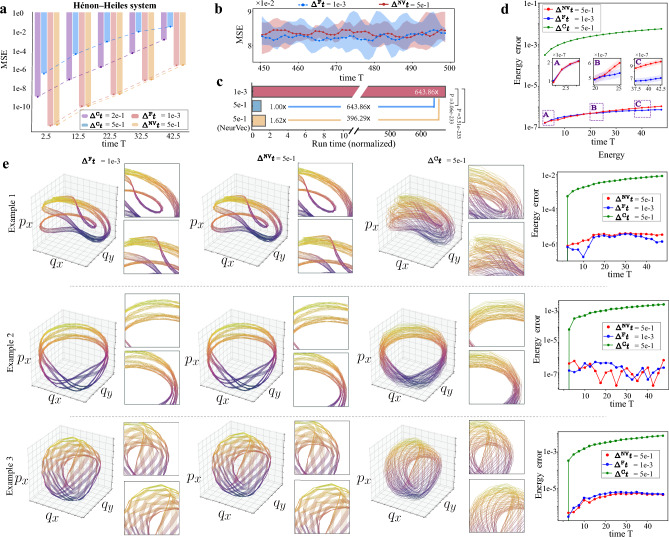
Performance comparison on the Hénon–Heiles system. (**a**) MSE with varied time on the time interval [0, 42.5] under different configurations (step size $\Delta ^{\textrm{C}}t = 5e-1$, $\Delta ^{\textrm{C}}t = 2e-1$, $\Delta ^{\textrm{F}}t=1e-3$, and NeurVec ($\Delta ^{\textrm{NV}}t = 5e-1$)). (**b**) MSE with varied time on the longer time interval [450, 500]. The upper and lower bounds of the light color indicate the maximal and minimal error, respectively. (**c**) The (normalized) runtime of the numerical solver with $\Delta ^{\textrm{F}}t=1e-3$, $\Delta ^{\textrm{C}}t=5e-1$, and NeurVec ($\Delta ^{\textrm{NV}}t=5e-1$). (**d**) Energy error with varied time on [0, 50]. (**e**) We provide three examples of trajectories projected on the coordinates $(q_x,q_y,p_x)$, and the corresponding energy error on [0, 50]. More examples can be found in the supplementary material.

1. Hénon–Heiles system
  The Hénon–Heiles system is a Hamiltonian system^60^ that describes the motion of a body around a center on the *x*-*y* plane. Let $(q_x,q_y)$ and $(p_x,p_y)$ denote the positions and momenta of a particle, respectively. The ODE is given by
  8 $$\begin{aligned} \frac{\text {d}}{\text {d}t}\left( q_x,q_y,p_x,p_y\right) = \left( p_x,p_y,-q_x-2\lambda q_xq_y, -q_y-\lambda (q_x^2-q_y^2)\right) . \end{aligned}$$
  The Hamiltonian (energy) function ${\mathcal {H}}$, defined by
  9 $$\begin{aligned} {\mathcal {H}}\left( q_x,q_y,p_x,p_y\right) = \frac{1}{2}(p_x^2+p_y^2)+\frac{1}{2}(q_x^2+q_y^2) + \lambda (q_x^2q_y-\frac{q_y^2}{3}), \end{aligned}$$
  must be conserved during the time evolution. This property is used as an additional metric to evaluate the accuracy of our method. We characterize the energy error by the absolute difference between the energy of the simulated trajectory and the initial energy. The datasets are generated with initial energy between $[\frac{1}{12}, \frac{1}{6}]$ and $-1< q_x < 1$, $-0.5< q_y < 1$ such that the equipotential curves of the system form an inescapable interior region and exhibit chaotic behavior^61^. The simulations are uniformly sampled every time interval $\eta = 5e-1$.
  We find that NeurVec ($\Delta ^{\textrm{NV}}t=5e-1$) vastly improves the accuracy of the ODE solvers, achieves almost the same accuracy as RK4 with $\Delta ^{\textrm{F}}t=1e-3$ on the time interval [0, 42.5] (Fig. 3a), and works well for a much larger time interval [450, 500] (Fig. 3c). Furthermore, NeurVec with $\Delta ^{\textrm{NV}}t=5e-1$ almost maintains the same system energy as does the reference method with $\Delta ^{\textrm{F}}t=1e-3$ (Fig. 3d). To illustrate the error correction capability of NeurVec, we visualize three trajectories of the first three components $(q_x,q_y,p_x)$ in Fig. 3e. For Examples 1–3 of Fig. 3e, NeurVec with $\Delta ^{\textrm{NV}}t=5e-1$ produces orbits similar to those of the reference method with $\Delta ^{\textrm{F}}t=1e-3$ while having an energy error of the same magnitude. Furthermore, the reference method with $\Delta ^{\textrm{C}}t=5e-1$ yields a larger energy error and pathwise difference. Integrating with $\Delta ^{\textrm{F}}t=1e-3$ over the time interval $\eta = 5e-1$ takes 500 steps, so it is not surprising that the runtime for $\Delta ^{\textrm{F}}t=1e-3$ is much larger than NeurVec with $\Delta ^{\textrm{NV}}t=5e-1$. Based on our test, NeurVec ($\Delta ^{\textrm{NV}}t=5e-1$) reaches more than 390$\times$ speedup over the reference method with $\Delta ^{\textrm{F}}t=1e-3$ (Fig. 3b).

**Figure 4:**
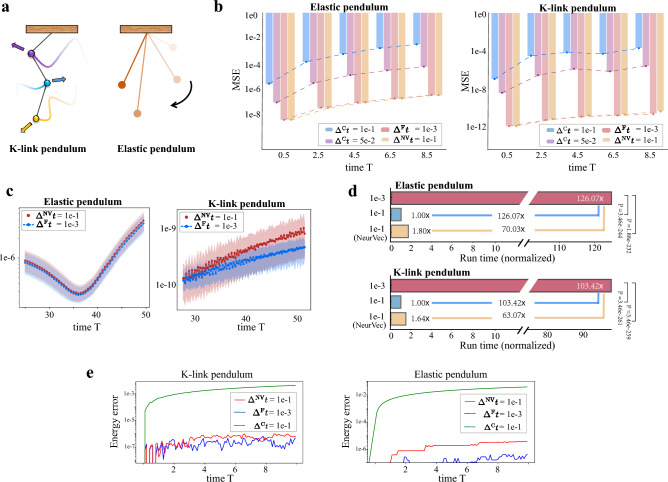
Performance comparison on the pendulum systems. (**a**) The pendulum systems. (**b**) MSE with varied time on the time interval [0, 8.5]. (**c**) MSE with varied time on the longer time interval [25, 50]. (**d**) The runtime comparison among the numerical solvers with $\Delta ^{\textrm{F}}t=1e-3$, $\Delta ^{\textrm{C}}t=1e-1$, and NeurVec ($\Delta ^{\textrm{NV}}t=1e-1$). (**e**) The energy error of two pendulum systems.
2. Elastic pendulum
  The elastic pendulum describes a point mass connected to a spring swinging freely (Fig. 4a), which may exhibit chaotic behavior under the force of gravity and spring^62^. We denote $\theta$ as the angle between the spring and the vertical line and *r* as the length of the spring. $\dot{\theta }$ and $\dot{r}$ correspond to the time derivative of $\theta$ and *r*, respectively. The motion of this system is governed by the ODE,
  10 $$\begin{aligned} \frac{\text {d}}{\text {d}t}\left( \theta ,r,\dot{\theta },\dot{r} \right) = \left( \dot{\theta },\dot{r},\frac{1}{r}(-g \sin \theta -\dot{\theta }\dot{r}),r\dot{\theta }^2-\frac{k}{m}(r-l_0)+g\cos \theta \right) , \end{aligned}$$
  where $k, m, l_0$, and *g* are spring constant, mass, original length, and gravity constant, respectively. The initial length of *r* is $r(0) = l_0 = 10$, $\dot{r}$ and $\dot{\theta }$ are initialized by constant 0, and $\theta$ is randomly sampled from the uniform distribution ${\mathcal {U}}([0,\frac{\pi }{8}])$. The simulations in the training and testing sets are uniformly sampled every time interval $\eta =1e-1$. NeurVec is trained on the simulation generated by $\Delta ^{\textrm{F}}t=1e-3$. NeurVec ($\Delta ^{\textrm{NV}}t=1e-1$) has accuracy of the same order as does $\Delta ^{\textrm{F}}t=1e-3$ on both short-term prediction (time interval [0, 8.5]) (Fig. 4b) and long-term prediction (time interval [25, 50]) (Fig. 4d). NeurVec ($\Delta ^{\textrm{NV}}t=1e-1$) is much faster than $\Delta ^{\textrm{F}}t=1e-3$ (*P* value $\ll$ 0.001 under two-sided t-tests), reaching about 70 × speedup (Fig. 4c).
3. *K* -link pendulum
  A *K*-link pendulum is a body suspended from a fixed point (Fig. 4a) with *K* rods and *K* bobs so that the body can swing back and forth under gravity^63^. The system exhibits chaotic behavior. For simplification, the length of each rod and the mass of each bob are set to 1, and the gravity constant *g* is set to 9.8. Let variables $\varvec{\theta }:= (\theta _1,\theta _2,\cdots ,\theta _K)$, where $\theta _i$ is the angle between the *i*th rod and the vertical axis. The system is governed by the ODE
  11 $$\begin{aligned} \frac{\text {d}}{\text {d}t}(\varvec{\theta },\dot{\varvec{\theta }}) = (\dot{\varvec{\theta }},\mathbf {A^{-1}}\textbf{b}). \end{aligned}$$
  Here $\textbf{b}=(b_1,b_2,\cdots ,b_K)$ and $b_i=-\sum _{j=1}^{K}\left[ c(i, j) \dot{\theta }_{j}^{2} \sin \left( \theta _{i}-\theta _{j}\right) \right] -(K-i+1) g\sin \theta _{i}$. $\textbf{A}$ is a $K \times K$ matrix with $\textbf{A}_{i, j}=c(i, j) \cos \left( \theta _{i}-\theta _{j}\right)$, where $c(i,j) = K - \max (i,j)+1$, for $i,j=1,\cdots ,K$. We use the example of a 2-link pendulum ($K=2$) to verify the advantage of NeurVec in terms of efficiency. The trajectory $(\varvec{\theta },\dot{\varvec{\theta }})$ is a 4-dimensional vector. The initial conditions $\varvec{\theta }(0)$ are randomly sampled on ${\mathcal {U}}[0,\pi /8]$, and $\dot{\varvec{\theta }}$ are set to zero (see the supplementary material). NeurVec ($\Delta ^{\textrm{NV}}t = 1e-1$) significantly improves the accuracy of $\Delta ^{\textrm{C}}t = 1e-1$, and achieves accuracy similar to that of RK4 ($\Delta ^{\textrm{F}}t = 1e-3$) for both short-term and long-term prediction (Fig. 4b and Fig. 4c). Moreover, NeurVec has over 63$\times$ speedup (Fig. 4d). Furthermore, Fig. 4e illustrates the energy error over time. We can see that NeurVec effectively conserves energy in simulations of a *K*-link pendulum and an elastic pendulum.

**Figure 5:**
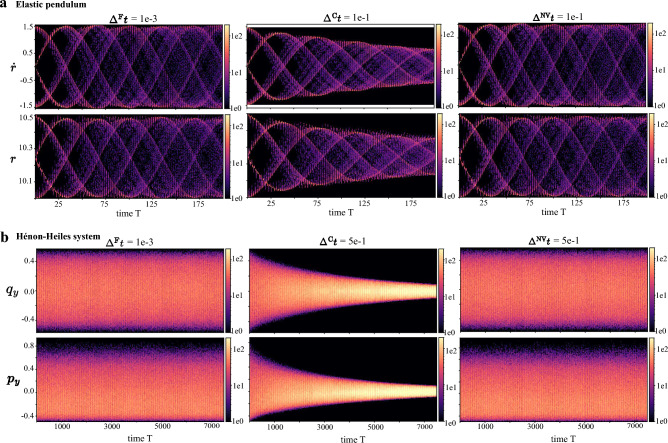
Time series histogram. We visualize the time series histogram of a test set for variables (**a**) *r* and $\dot{r}$ in the elastic pendulum (10) and (**b**) $q_y$ and $p_y$ in the Hénon–Heiles system (8). The color represents the number count (the lighter color and the larger frequency). The solutions generated by the solver with coarse step size exhibits a trend of convergence to a specific value, while solutions of the solver with fine step size are distributed within a range, and NeurVec with coarse step size produces a histogram visually identical with that of the solver with fine step size. This result shows that NeurVec has a more accurate solution than does the solver with fine step size.

## Analysis

In this section we provide further analysis of the performance of NeurVec from three aspects: (1) NeurVec maintains the statistic of the solutions from large-scale simulation, which is crucial for constructing reliable models; (2) NeurVec learns the leading error term of the numerical solver, which enables a more accurate estimation for each step; and (3) we compare the evaluation time and solution error among solver with fine or coarse step size and NeurVec.

### Maintaining solution statistics in large-scale simulations

We validate the performance of NeurVec on producing consistent statistical observations for ensemble forecasting. The ability to enable a large step size for a set of sampled initial conditions is critical for real applications such as weather forecasting. We visualize the time series histogram of the testing set for variables (**a**) *r* and $\dot{r}$ in the elastic pendulum (10) and (**b**) $q_y$ and $p_y$ in the Hénon–Heiles system (8) in Fig. 5. The time series histogram is generated by dividing axes into $800\times 100$ bins and counting the curves that cross the bins.

For the elastic pendulum, we find that starting from a time $T\ge 25$, the statistical difference of $\dot{r}$ and *r* between $\Delta ^{\textrm{F}}t=1e-3$ and $\Delta ^{\textrm{C}}t=1e-1$ becomes larger. When the step size is $\Delta ^{\textrm{F}}t=1e-3$, $\dot{r}$ exhibits periodic behavior, which is in accordance with the periodic variation of the spring during its extend-retract. However, let $\Delta ^{\textrm{C}}t=1e-1$, $\dot{r}$ and *r* show a trend of approaching specific values, and the change range gradually narrows. On the other hand, the simulations with NeurVec ($\Delta ^{\textrm{NV}}t=1e-1$) have a pattern smilar to that of $\Delta ^{\textrm{F}}t=1e-3$. We have a similar observation for $q_y$ and $p_y$ in Hénon–Heiles system (Fig. 5b). Therefore, we conclude that NeurVec produces more accurate solutions compared with the reference method with large step size, enabling better and more consistent statistical observation.

### Learnability and generalizability

In Eq. (4), we start from the mathematical expression of the error term $\text {err}_n(k,\Delta t, \textbf{u}(t))$ in the form of $\frac{1}{n!} \frac{\text {d}^n}{\text {d}t^n}\textbf{u}(t)\cdot [k\Delta t]^n$, and consider using a neural network to approximate the sum of all error terms. However, neural networks are generally regarded as black box functions with a lack of interpretability. Therefore, in order to explain the good performance of NeurVec and confirm the learnability of the error terms, in this section, we explore and visualize what the neural network in NeurVec learns. We consider solving a 1-link pendulum with the Euler method. Our consideration for testing NeurVec on this system is based on the following motivations. First, the dimension of the state is 2, which facilitates error visualization on phase space. Second, we derive the error term of the Euler method explicitly through the Taylor formula:

12 $$\begin{aligned} \textbf{u}(t+\Delta t) - \left( \textbf{u}(t) + \textbf{f}(\textbf{u})\Delta t \right) = \frac{1}{2}(\nabla \textbf{f}) \textbf{f}(\textbf{u})\Delta t^2 +{\mathcal {O}}(\Delta t^3), \end{aligned}$$

where $\nabla \textbf{f}$ is the Jacobian matrix of $\textbf{f}$. The second-order term $\frac{1}{2}(\nabla \textbf{f}) \textbf{f}(\textbf{u})\Delta t^2$ is the leading error term of the Euler method, which is supposed to be captured by NeurVec from data of fine step size.

**Figure 6:**
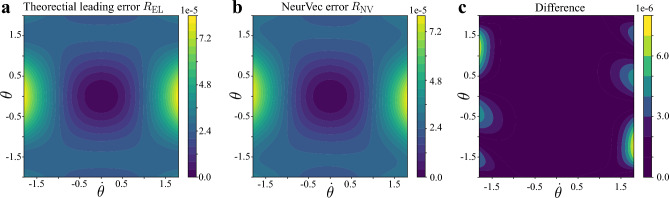
Numerical error visualization on the phase space of 1-link pendulum. (**a**) The square sum of leading (second-order) error term of the Euler method, denoted by $R_{\textrm{EL}}$. The error is calculated by using the true dynamics *f*. (**b**) The square sum norm of error compensation learned by NeurVec, denoted by $R_{\textrm{NV}}$. (**c**) The difference between the leading error term and NeurVec.

Denote $R_{\text {NV}}(\textbf{u}):=\left\| \text {NeurVec}(\textbf{u})\right\| _2^2$ as the norm of error learned by NeurVec and $R_{\text {EL}}(\textbf{u}):=\left\| \frac{1}{2}(\nabla \textbf{f}) \left( \textbf{f}(\textbf{u}\right) \Delta t^2\right\| _2^2$ as the norm of the leading error term of the Euler method. To train NeurVec for the Euler method, we generate the dataset by randomly sampling the initial conditions of $\theta$ and $\dot{\theta }$ from uniform distributions ${\mathcal {U}}([0,\pi /2])$ and ${\mathcal {U}}([0,0.5])$, respectively, and then use the Euler method to simulate the data with $dt = 1e-3$. We train NeurVec with coarse step size $dt=1e-1$.

We found that the learned error $R_{\text {NV}}$ (Fig. 6a) is visually consistent with the leading error $R_{\text {EL}}$ of the Euler method (Fig. 6b). The squared difference $R_{\text {Diff}}=\left\| \frac{1}{2}(\nabla \textbf{f}) \left( \textbf{f}(\textbf{u}\right) \Delta t^2-\text {NeurVec}(\textbf{u})\right\| _2^2$ is up to order ${\mathcal {O}}(10^{-6})$, and only a small part of the difference near the boundary is relatively large (Fig. 6c). The relatively significant differences near the boundary may be attributed to the limited availability of data that encompasses those boundary regions. (see the supplementary material for details). Through the training data of high accuracy, NeurVec captured the leading error term of the numerical solver. NeurVec may even capture the higher-order error terms, which enables the use of a coarse step size. Furthermore, we can analyze the generalization ability of NeurVec from Fig.6. On one hand, the results displayed in Fig. 6c demonstrate that NeurVec is capable of generalizing well on a continuous phase space, even when trained using limited and discrete data. This suggests that NeurVec exhibits good interpolation generalization abilities. On the other hand, it is noteworthy that the initial conditions of our training data were sampled from ${\mathcal {U}}([0,\pi /2])$ and ${\mathcal {U}}([0,0.5])$, and the testing range shown in Fig. 6 shows the neural network’s effective approximation of the error term and implicit prior information, indicating a certain degree of extrapolation capability. Such findings suggest that NeurVec demonstrates a promising potential for generalizing beyond the training data.

### Theoretical analysis

We analyze the runtime and the global error in the solution approximated with NeurVec. Let $0=t_0<t_1<\cdots <t_{pk}=T$ be uniform points on [0, *T*] and $\Delta t=\frac{T}{pk}$.

We compare the runtime of fine and coarse step size. If the step size $\Delta t$ is used, then the number of steps for integration is $\frac{T}{\Delta t}$. If the step size is $k\Delta t$, then the number of steps needed is $\frac{T}{k\Delta t}$. $\epsilon$ is the ratio of the runtime of NeurVec to that of scheme *S* for one step. Hence, when NeurVec is used to integrate with step size $k\Delta t$, we need $\epsilon \times 100\%$ extra time for each step; and the time becomes ${\mathcal {O}}(\frac{T(1+\epsilon )}{k\Delta t})$.

Next we study the error of solvers with fine or coarse step size. For simplification, we focus on the Euler solver and characterize the global discretization error (difference between the true solution and the estimated solution) at the time *T*. When the Euler scheme is used,

13 $$\begin{aligned} \textbf{u}_{n+1} = \textbf{u}_{n} + \Delta t \textbf{f}(\textbf{u}_{n}), \quad \textbf{u}_0 = \textbf{c}_0, \quad n=0,1,\cdots , kp-1. \end{aligned}$$

#### Proposition 0.1

We assume that (1) $\textbf{f}$ is Lipschitz continuous with Lipschitz constant *L* and (2) the second derivative of the true solution $\textbf{u}$ is uniformly bounded by $M>0$, namely., $\Vert \textbf{u}''\Vert _\infty \le M$ on [0, *T*]. Then, using (13), we have

$$\begin{aligned} |\textbf{u}_{kp}-\textbf{u}(T)|\le \frac{M\exp (2TL)}{2L}\Delta t . \end{aligned}$$

For the proof, see the work of Atkinson et al.^64^. Proposition 0.1 shows that the Euler method converges linearly. The error is ${\mathcal {O}}(\Delta t)$ when the step size $\Delta t$ is used. Similarly, the error becomes ${\mathcal {O}}(k\Delta t)$ when the step size is $k\Delta t$. We next derive the error for the Euler method with step size $k\Delta t$ using NeurVec. The iterative formula is given by

14 $$\begin{aligned} \hat{\textbf{u}}_{k(n+1)} = \hat{\textbf{u}}_{kn} + f(\hat{\textbf{u}}_{kn}) (k\Delta t) + \text {NeurVec}(\hat{\textbf{u}}_{kn};{\Theta }), \quad \hat{\textbf{u}}_0 = c_0, \quad n=0,1,\cdots , p-1. \end{aligned}$$

We can use the following loss function to identify the learnable parameter $\Theta$ in NeurVec. $V_n$ denotes the residual error for each term.

15 $$\begin{aligned} \text {LS} = \frac{1}{p}\sum _{n=0}^{p-1}\big \Vert \frac{\textbf{u}_{k(n+1)}-\textbf{u}_{kn}}{k\Delta t} - \textbf{f}(\textbf{u}_{kn}) - \frac{\text {NeurVec}(\textbf{u}_{kn};{\Theta })}{k\Delta t}\Vert _2^2:=\frac{1}{p}\sum _{n=0}^{p-1}\Vert V_n\Vert _2^2 \end{aligned}$$

In the next theorem we characterize the error of NeurVec ($k\Delta t$) by the quality of the training data and the neural network training error. In addition to the assumption in Proposition 0.1, we assume NeurVec is Lipschitz continuous and the Lipschitz constant is of order $k\Delta$. This assumption is reasonable based on the following motivation. According to Taylor expansion $v(t+\Delta t)=v(t)+v'(t)\Delta t+o(\Delta t)$, from our objective we expect that $\text {NeurVec}\sim o(k\Delta t)$.

#### Theorem 0.1

Assumptions (1) and (2) in Proposition 0.1 hold. In addition, we assume that $\text {NeurVec}$ is Lipschitz continuous with Lipschitz constant $k\Delta t L_{NV}$, which is independent of $\varvec{\theta }$. Then the error is

16 $$\begin{aligned} |\hat{\textbf{u}}_{kp}-\textbf{u}(T)|\le \frac{M\exp (2TL)}{2L}\Delta t + \frac{\sqrt{T}\exp (T(L+L_{NV}))}{\sqrt{L+L_{NV}}}(\textrm{LS})^{\frac{1}{2}.} \end{aligned}$$

The first term in the right-hand side of (16) comes from the error of the training data, Euler simulation with step size $\Delta t$, while the second term is the training error of NeurVec. A series of works^65–67^ utilize a neural tangent kernel to prove the global convergence of a neural-network-based least squares method. Under the assumptions of training data distribution, when the width of one hidden layer network is sufficiently large, gradient descent converges to a globally optimal solution for the quadratic loss function. We might assume that the training error $\text {LS}\rightarrow 0$ as the increasing update iteration. Then in (16), $|\hat{\textbf{u}}_{kp}-\textbf{u}(T)|\sim {\mathcal {O}}(\Delta t)$.

## Discussion

To address the speed-accuracy trade-off in large-scale simulations of dynamical systems, we proposed NeurVec, a deep learning based corrector in the numerical solver, which can compensate for the error caused by the use of coarse step size for numerical solvers. Through extensive experiments and preliminary theoretical evidence, we show that NeurVec is general and can be applied to widely used explicit integration methods and learn the error distribution through simulations with fine step size. However, there are still some limitations while using NeurVec.

**Figure 7:**
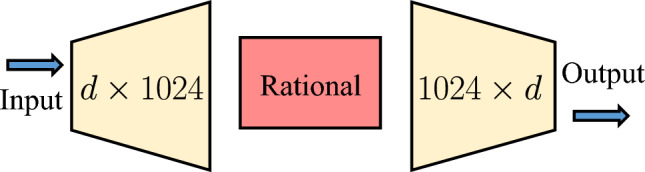
Neural network structure in NeurVec. NeurVec consists of two linear transformations layers (yellow) and one nonlinear activation function layer (red), which is a feed-forward neural network of one hidden layer ($L=1$) with the width $N_1=1024$.

Notice that, according to the analysis in Table 1, NeurVec can achieve fast simulation, i.e., a speedup of ${\mathcal {O}}(k/(1+\epsilon ))$ times, under the condition that, after the neural network is trained, it can maintain the same accuracy as the training data at a sufficiently large step size. However, there exist some complex differential equations, such as ultra-high dimensional dynamical systems, may not satisfy this condition and prevent the term $\frac{\sqrt{T}\exp (T(L+L_{NV}))}{\sqrt{L+L_{NV}}}(\textrm{LS})^{\frac{1}{2}}$ in Eq. (16) from converging to 0, which would make NeurVec not necessarily achieve the target accuracy ${\mathcal {O}}(\Delta t)$. Specifically, we consider a general high-dimensional dynamical system with dimension *d*, and a shallow network structure with a width of $N_1$ as shown in Fig.7 as an example. In addition, Lu et al^68^ reveal that for an infinitely deep network with input dimension *d* and some mild assumptions, it can achieve an arbitrary accuracy while the width of network is at least ${\mathcal {O}}(d)$. Therefore, the dimension of differential equations that NeurVec can currently handle will not exceed ${\mathcal {O}}(N_1)$, and in practice, since the neural network depth is limited, the available dimension may be much lower than ${\mathcal {O}}(N_1)$. To improve the accuracy, the easiest way is to widen the neural network as much as possible or consider more advanced learning architectures and training algorithms, for example, an attention mechanism^69–71^, neural network structure search^72–74^, and large-scale pretrained models^75^. However, these improvements will increase $\epsilon$, and the speedup ratio ${\mathcal {O}}(k/(1+\epsilon ))$ will become relatively small at this time, even accelerated simulations cannot be achieved while $\epsilon$ is large enough.

In Table 2, we consider the Sping-chain system to explore the impact of ultra-high dimensional dynamical systems on NeurVec. In this paper, we set $$N_1 = $$ 1024, the experimental results show that as the dimension increases, the accuracy of NeurVec becomes harder to maintain, especially in the case that the dimension close to $$\kappa $$, which is consistent with our analysis.

**Table 2:** The impact on accuracy (MSE at evaluation time $T=20$) under the ultra-high dimension systems. All experimental settings are following the experiments in Fig.2. The “Dim” is the dimension of the dynamical system (Spring-chain). We also consider the student t-test with a significant level of 0.05 for NeurVec. If the p-value P < 0.05, there exists a significant difference between NeurVec and the corresponding traditional numerical solvers and it also means NeurVec can not maintain consistent accuracy.

Method	Step size	Dim = 300	*P* < 0.05?	Dim = 500	*P* < 0.05?	Dim = 1000	*P* < 0.05?
Euler	1e−3	2.58e−2	–	7.32e−2	–	5.48e−1	–
Euler+NeurVec	2e−1	2.58e−2	×	7.33e−2	×	5.54e−1	×
RK4	1e−3	4.86e−7	–	4.64e−6	–	6.42e−5	–
RK4+NeurVec	2e−1	4.84e−7	×	1.05e−5	$\checkmark$	2.61e−3	$\checkmark$

In addition, we may extend NeurVec in several ways.

- *Generalizing NeurVec* We implemented NeurVec using the simulation data with fixed system parameters and fixed step size. Recently, the neural network, as a universal approximator, shows promising results on learning the nonlinear continuous operator. Motivated by operator learning^45,50^, we may add additional dimensions to the input of NeurVec, such as the step size for integration and the physical parameters in the system. Then NeurVec can be trained with more diverse simulation data, such that NeurVec can be used with different *dt* for systems with varied physical parameters (such as $\varvec{k}$ and $\varvec{m}$ in the spring-chain system (7)).
- *Continual model update* Neural networks may sometimes have inaccurate predictions when encountering abnormal situations. Therefore, we may need to maintain and update NeurVec regularly to learn from new data. The simplest strategy is to retrain the network from scratch, but it needs considerable computing resources to train and memory resources to store the data. To address such a problem, we can fine-tune NeurVec via incremental learning^76^ for a small amount of newly collected training data to achieve low-cost model updates.
- *Numerical solvers and cutting-edge problems* In this paper, we consider four forward numerical solvers and four kinds of ODE problems. NeurVec can be extended to other types of numerical solvers, such as backward methods and implicit methods. These methods are mainly aimed at improving the simulation accuracy, but the simulation cost for one step may be large. For more intricate cutting-edge problems, NeurVec may require further tuning of training parameters, neural network architecture, and data processing, as explored in Refs.^47,49,77^. Nevertheless, the NeurVec framework we introduce is general and easily extendable. We believe NeurVec has the potential to be adopted for more complex dynamical systems.

## Methods

### Datasets

We summarize the simulated training and testing datasets used in the main text in Table 3. For each dataset we integrate with the step size $\delta$ using the numerical solver over *N* random initializations. We obtain the discrete solutions every $\delta$ up to the model time *T*. Next, we sample the solution every time interval $\eta$ ($\eta$ is a multiple of $\delta$).

**Table 3:** Summary of the simulated datasets used in the main text.

Problem	Type	Dim	Num	Step size $\delta$	Method	Duration *T*
Spring-chain (Euler)	Train	40	60k	1e−3	Euler	20
Spring-chain (Improved Euler)	Train	40	60k	1e−3	Improved Euler	20
Spring-chain (RK3)	Train	40	60k	1e−3	RK3	20
Spring-chain (RK4)	Train	40	60k	1e−3	RK4	20
Spring-chain	Test	40	10.5k	1e−4	RK4	20
1-link pendulum	Train	2	1k	1e−3	RK4	10
2-link pendulum	Train	4	300k	1e−3	RK4	10
2-link pendulum	Test	4	7k	1e−4	RK4	10
Hénon–Heiles	Train	4	100k	1e−3	RK4	50
Hénon–Heiles	Test	4	70k	1e−4	RK4	50
Elastic pendulum	Train	4	300k	1e−3	RK4	50
Elastic pendulum	Test	4	14k	1e−4	RK4	50

### Numerical solvers

We introduce four numerical solvers used in our paper: the Euler method, improved Euler method, and 3rd- and 4th-order Runge–Kutta methods. These solvers have different $S(f, u_{n}, \Delta t_n)$ in the iterative formula (2). (1) The Euler method can be written as

17 $$\begin{aligned} S(\textbf{f}, \textbf{u}_{n}, \Delta t_n) = \Delta t_n \textbf{f}(\textbf{u}_{n}). \end{aligned}$$

It has an explicit geometric interpretation—it uses a series of line segments to approximate the solution of the equation. It is first-order accurate since its local truncation error is ${\mathcal {O}}(\Delta t^2)$ and the global error is ${\mathcal {O}}(\Delta t)$.

(2) The improved Euler method^78,79^ can be written as

18 $$\begin{aligned} S(\textbf{f}, \textbf{u}_{n}, \Delta t_n) =\frac{\Delta t_n }{2}[ \textbf{f}(\textbf{u}_{n}) + \textbf{f}(\textbf{u}_{n} + \Delta t_n \textbf{f}(\textbf{u}_{n}))]. \end{aligned}$$

The improved Euler method is a numerical method that uses an implicit trapezoidal formulation to improve the accuracy of the Euler method. Specifically, it first takes a one-step Euler method to obtain ${\tilde{u}}_{n+1} = u_{n} + \Delta t_n f(u_{n})$ and then uses the implicit trapezoidal formula to obtain $u_{n+1} = u_{n} + \frac{\Delta t_n }{2}[f(u_{n}) + f({\tilde{u}}_{n+1})]$. Even though the improved Euler method requires more computation compared with the Euler method, it has a higher accuracy with a local error of ${\mathcal {O}}(\Delta t^3)$. (3) The *m*th-order Runge–Kutta method can be written as

19 $$\begin{aligned} S(\textbf{f}, \textbf{u}_{n}, \Delta t_n) = \Delta t_n \sum _{i=1}^m \lambda _iK_i. \end{aligned}$$

For the 3rd-order RK method, the number of stages $m=3$, the coefficients $\lambda _1 = \lambda _3 = \frac{1}{6}$ and $\lambda _2 = \frac{2}{3}$, and the update rule $K_1 = \textbf{f}(\textbf{u}_{n}), K_2 = \textbf{f}(\textbf{u}_{n}+\frac{\Delta t_n}{2}K_1)$ and $K_3 = \textbf{f}(\textbf{u}_{n} - \Delta t_nK_1+2\Delta t_nK_2)$. For the 4th-order RK method, $m=4$, $\lambda _1 = \lambda _4 = \frac{1}{6}$ and $\lambda _2 = \lambda _3 = \frac{1}{3}$. The update rule $K_1 = \textbf{f}(\textbf{u}_{n}), K_2 = \textbf{f}(\textbf{u}_{n}+\frac{\Delta t_n}{2}K_1), K_3 = \textbf{f}(\textbf{u}_{n}+\frac{\Delta t_n}{2}K_2)$ and $K_4 = \textbf{f}(\textbf{u}_{n}+\Delta t_nK_3)$. Runge–Kutta methods, especially the 4th-order Runge–Kutta method, are widely used in engineering and natural sciences. The Euler method and improved Euler method can also be seen as special Runge–Kutta methods. When using the larger order *m* in Eq. (19), we need to compute iteratively a series of $K_i,i=1,2, \ldots, m$, increasing the computation cost for each step.

### Implementation details of NeurVec

We use a fully connected neural network to model NeurVec in Eq. (5). The fully connected feed-forward neural network is the composition of *L* nonlinear functions:

20 $$\begin{aligned} \phi (\textbf{x};\varvec{\theta }):=\mathbf {W_a} \circ \textbf{h}_L \circ \textbf{h}_{L-1} \circ \cdots \circ \textbf{h}_{1}(\textbf{x}), \end{aligned}$$

where $\textbf{h}_{\ell }(\textbf{x})=\sigma \left( \textbf{W}_\ell \textbf{x} + \textbf{b}_\ell \right)$ with $\textbf{W}_\ell \in {\mathbb {R}}^{N_{\ell }\times N_{\ell -1}}$, $\textbf{b}_\ell \in {\mathbb {R}}^{N_\ell }$ for $\ell =1,\dots ,L$, $\mathbf {W_a}\in {\mathbb {R}}^{d\times N_L}$, $\textbf{x}\in R^{d\times N}$, $\sigma$ is a nonlinear activation function, for example, a rectified linear unit (ReLU) $\sigma (x)=\max \{x,0\}$ or hyperbolic tangent function $\tanh (x)$, *d* is the dimension of the state, and *N* is the batch size. Each $\textbf{h}_\ell$ is referred to as a hidden layer, where $N_\ell$ is the width of the $\ell$th layer. In this formulation, $\varvec{\theta }:=\{\mathbf {W_a},\,\textbf{W}_\ell ,\,\textbf{b}_\ell :1\le \ell \le L\}$ denotes the set of all parameters in $\phi$, which uniquely determines the underlying neural network. In our implementation (Fig. 7), the feed-forward neural network is of one hidden layer ($L=1$) with the width $N_1=1024$. The activation function used is the rational activation function^58^ defended by

21 $$\begin{aligned} \frac{a_3x^3+a_2x^2+a_1x^1+a_0}{b_2x^2+b_1x^1+b_0}, \end{aligned}$$

where $a_i,0\le i\le 3$ and $b_i,0\le i\le 2$ are initialized by constants $a_0=0.0218$, $a_1 = 0.5000$, $a_2 = 0.5957$, $a_3 = 1.1915$, $b_0 = 1.0000$, $b_1 = 0.0000$, $b_2 = 2.3830$, respectively. The parameters in $\textbf{W}_a$ and $\textbf{W}_\ell$ are initialized from ${\mathcal {U}}[-1/\sqrt{N_0},1/\sqrt{N_0}]$ and ${\mathcal {U}}[-1/\sqrt{N_1},1/\sqrt{N_1}]$, respectively. We optimize the $\phi$ for 500 epochs with the Adam optimizer. Moreover, we use the mean square error as the objective function in Eq. (6), and we set the initial learning rate to 1e-3.

### Supplementary Information

Supplementary Information.

## Data Availability

The synthesized data for the different complex dynamical systems and source codes for training and testing results are available at the online data warehouse: https://github.com/dedekinds/NeurVec. The source codes are released under MIT license.
