# Supplementary material for: On fast simulation of dynamical system with neural vector enhanced numerical solver
Source: Sci Rep. 2023 Sep 14;13:15254. doi: 10.1038/s41598-023-42194-y (PMC10502038; doi:10.1038/s41598-023-42194-y)
Supplement: Supplementary file 1 — Supplementary Information. [file 41598_2023_42194_MOESM1_ESM.pdf]

Supplementary

Proof of the Theorem

Proof. We denote $E_n := \hat{\mathbf{u}}_{kn} - \mathbf{u}_{kn}$. Then by triangle inequality and Proposition 0.1 in the main text, we have

$$|\hat{\mathbf{u}}_{kp} - \mathbf{u}(T)| \leq |\mathbf{u}_{kp} - \mathbf{u}(T)| + |\hat{\mathbf{u}}_{kp} - \mathbf{u}_{kp}| = E_p + \frac{M\exp(2TL)}{2L}\Delta t. \tag{22}$$

Next we estimate the error E_p. We have

$$\begin{aligned}\hat{\mathbf{u}}_{k(n+1)} - \mathbf{u}_{k(n+1)} &= \hat{\mathbf{u}}_{kn} + \mathbf{f}(\hat{\mathbf{u}}_{kn})(k\Delta t) + \mathrm{NeurVec}(\hat{\mathbf{u}}_{kn};\boldsymbol{\theta}) - \mathbf{u}_{k(n+1)} \\ &= \hat{\mathbf{u}}_{kn} - \mathbf{u}_{kn} + \big(\mathbf{f}(\hat{\mathbf{u}}_{kn}) - \mathbf{f}(\mathbf{u}_{kn})\big)(k\Delta t) + \mathrm{NeurVec}(\hat{\mathbf{u}}_{kn};\boldsymbol{\theta}) - \mathrm{NeurVec}(\mathbf{u}_{kn};\boldsymbol{\theta}) - (k\Delta t)V_n.\end{aligned}$$

Then using assumption (1),

$$\begin{aligned}|\hat{\mathbf{u}}_{k(n+1)} - \mathbf{u}_{k(n+1)}| &\leq |\hat{\mathbf{u}}_{kn} - \mathbf{u}_{kn}| + L|\hat{\mathbf{u}}_{kn} - \mathbf{u}_{kn}|(k\Delta t) + k\Delta t L_{NV}|\hat{\mathbf{u}}_{kn} - \mathbf{u}_{kn}| + (k\Delta t)|V_n| \\ &= (1 + k\Delta tL + k\Delta tL_{NV})|\hat{\mathbf{u}}_{kn} - \mathbf{u}_{kn}| + (k\Delta t)|V_n|.\end{aligned}$$

we denote the constant $(1 + k\Delta tL + k\Delta tL_{NV})$ as w. We rewrite the above inequality as $|E_{n+1}| \leq w|E_n| + (k\Delta t)|V_n|$. Then

$$\begin{aligned}|E_{n+1}| &\leq w|E_n| + (k\Delta t)|V_n| \\ &\leq w(w|E_{n-1}| + (k\Delta t)|V_{n-1}|) + (k\Delta t)|V_n| = w^2|E_{n-1}| + w(k\Delta t)|V_{n-1}| + (k\Delta t)|V_n| \\ &\leq \cdots \\ &\leq w^{n+1}|E_0| + (k\Delta t)\sum_{i=0}^{n} w^i|V_{n-i}| = (k\Delta t)\sum_{i=0}^{n} w^i|V_{n-i}|,\end{aligned}$$

where $E_0 = 0$ as $E_0 = \hat{\mathbf{u}}_0 - \mathbf{u}_0 = \mathbf{c}_0 - \mathbf{c}_0 = 0$. By the Cauchy inequality,

$$|E_p| \leq (k\Delta t)(\sum_{i=0}^{p-1} w^{2i})^{\frac{1}{2}}(\sum_{i=0}^{p-1}|V_{p-1-i}|^2)^{\frac{1}{2}} = (k\Delta t)(\frac{w^{2p}-1}{w^2-1})^{\frac{1}{2}}(p\mathrm{LS})^{\frac{1}{2}}.$$

Note that $\frac{w^{2p}-1}{w^2-1} \leq \frac{(1+k\Delta tL+k\Delta tL_{NV})^{2p}}{k\Delta tL+k\Delta tL_{NV}} \leq \frac{\exp(2pk\Delta t(L+L_{NV}))}{k\Delta tL+k\Delta tL_{NV}} = \frac{\exp(2T(L+L_{NV}))}{k\Delta t(L+L_{NV})}$. We obtain the bound by

$$|E_p| \leq (k\Delta t)\frac{\exp(T(L+L_{NV}))}{\sqrt{k\Delta t(L+L_{NV})}}(p\mathrm{LS})^{\frac{1}{2}} = \frac{\sqrt{T}\exp(T(L+L_{NV}))}{\sqrt{L+L_{NV}}}(\mathrm{LS})^{\frac{1}{2}}.$$

Combining (22), we end our proof. □

Details of the acceleration.

Runtime

We presented the normalized runtime results of different configurations in the main text. Here we provide details about the implementation and the exact runtime values.

We divide the testing dataset of each problem (Table 3 in the main text) equally into 70 batches. Then, we simulate each batch sequentially on a single GeForce RTX 3080 GPU and record their inference time at each run. In order to mitigate the GPU from overheating, a 10-second pause is executed between every two runs. The mean clock time and its standard derivation (std) are reported in Table S1.

Problems	Method	Step size	Time-mean (sec.)	Time-std
Spring-chain	Euler	2e-1	0.099	0.002
Spring-chain	Euler	1e-3	18.510	0.361
Spring-chain	Euler+NeurVec	2e-1	0.124	0.011
Spring-chain	Improved Euler	2e-1	0.172	0.002
Spring-chain	Improved Euler	1e-3	38.891	2.831
Spring-chain	Improved Euler+NeurVec	2e-1	0.237	0.030
Spring-chain	RK3	2e-1	0.260	0.002
Spring-chain	RK3	1e-3	57.921	0.416
Spring-chain	RK3+NeurVec	2e-1	0.348	0.028
Spring-chain	RK4	2e-1	0.359	0.002
Spring-chain	RK4	1e-3	73.012	2.261
Spring-chain	RK4+NeurVec	2e-1	0.463	0.040
Hénon-Heiles	RK4	5e-1	0.009	0.001
Hénon-Heiles	RK4	1e-3	5.984	0.097
Hénon-Heiles	RK4+NeurVec	5e-1	0.015	0.001
2-link pendulum	RK4	1e-1	0.269	0.004
2-link pendulum	RK4	1e-3	27.801	0.280
2-link pendulum	RK4+NeurVec	1e-1	0.441	0.061
Elastic pendulum	RK4	1e-1	0.056	0.001
Elastic pendulum	RK4	1e-3	7.011	0.094
Elastic pendulum	RK4+NeurVec	1e-1	0.100	0.059

Table S1: Comparison of mean runtime and its standard deviation over 70 batches of simulation.

Statistical test

We statistically validate the acceleration performance of NeurVec. We collected the runtime results of solver with fine step size and NeurVec with coarse step size as in Table S1; each of them contains 70 samples. In the t-test, the null hypothesis is that the runtimes of solver with fine step size and NeurVec with coarse step size are identical. We use P_1 to denote the P-value of the two-sided t-test and P_2 to denote the P-value of the Welch's t-test. These two t-tests have the same statistics but for different situations. We use the two-sided t-test if the the pair of the runtimes have the same variance. Otherwise, we use Welch's t-test. In Table S2, all the P_1 and P_2 are much smaller than $1e-3$, indicating that the null hypothesis is rejected and the pair of runtime samples are different. In other words, NeurVec can accelerate the simulation with statistical significance.

Problem	Method	Setting1	Setting2	Statistics	P_1 ($P_1 \ll$1e-3?)	P_2 ($P_2 \ll$1e-3?)
Spring-chain	Euler	1e-3	2e-1	461.97	5.59e-222 ✓	3.55e-122 ✓
Spring-chain	Euler	1e-3	2e-1+NeurVec	461.07	7.30e-222 ✓	2.44e-122 ✓
Spring-chain	Imp-Euler	1e-3	2e-1	125.34	4.87e-144 ✓	3.88e-83 ✓
Spring-chain	Imp-Euler	1e-3	2e-1+NeurVec	125.11	6.24e-144 ✓	4.22e-83 ✓
Spring-chain	RK3	1e-3	2e-1	1252.16	1.01e-281 ✓	4.73e-152 ✓
Spring-chain	RK3	1e-3	2e-1+NeurVec	1246.92	1.80e-281 ✓	2.41e-153 ✓
Spring-chain	RK4	1e-3	2e-1	291.81	1.78e-194 ✓	2.08e-108 ✓
Spring-chain	RK4	1e-3	2e-1+NeurVec	291.32	2.25e-194 ✓	2.00e-108 ✓
Hénon-Heiles	RK4	1e-3	5e-1	557.53	3.06e-233 ✓	7.91e-128 ✓
Hénon-Heiles	RK4	1e-3	5e-1+NeurVec	556.97	3.51e-233 ✓	8.48e-128 ✓
2-pendulum	RK4	1e-3	1e-1	888.86	3.46e-261 ✓	8.85e-142 ✓
2-pendulum	RK4	1e-3	1e-1+NeurVec	859.68	3.46e-259 ✓	2.30e-154 ✓
Elastic pendulum	RK4	1e-3	1e-1	669.28	3.46e-244 ✓	2.63e-133 ✓
Elastic pendulum	RK4	1e-3	1e-1+NeurVec	550.22	1.88e-232 ✓	7.35e-208 ✓

Table S2: The statistical test for different pair run time results. P_1 is the p-value of the two-sided t-test and P_2 is the p-value of the Welch's t-test.

Summary of the initialization of different systems.

m	0.900	0.938	0.925	0.787	0.667	k	3.900	3.508	5.651	5.533	3.664	
	1.348	0.776	0.692	0.941	0.538		4.373	2.555	5.239	6.024	6.942	
	1.215	0.821	1.121	0.875	1.456		5.073	3.941	4.505	4.744	3.805	
	1.111	1.125	1.431	0.663	1.222		4.848	3.477	3.405	2.499	4.735	4.891

Table S3: The values of m and k in spring-chain systems: m and k are obtained by random and independent sampling.

**Task**	**Variable**	**Dim**	**Type**	**Range**
Spring-chain	$\mathbf{p}$	20	Uniform random	$[-2.5, 2.5]^{20}$
Spring-chain	$\mathbf{q}$	20	Uniform random	$[-2.5, 2.5]^{20}$
Hénon–Heiles	q_x	1	Uniform random	[-1,1]
Hénon-Heiles	q_y	1	Uniform random	[-0.5,1]
Hénon–Heiles	p_x	1	Uniform random	[-1,1]
Hénon–Heiles	p_y	1	Uniform random	[-1,1]
Elastic pendulum	θ	1	Uniform random	$[0, \pi/8]$
Elastic pendulum	r	1	Constant	10
Elastic pendulum	$\dot{\theta}$	1	Constant	0
Elastic pendulum	$\dot{r}$	1	Constant	0
Elastic pendulum	l_0	1	Constant	10
Elastic pendulum	g	1	Constant	9.8
Elastic pendulum	k	1	Constant	40
Elastic pendulum	m	1	Constant	1
2-pendulum	θ	2	Uniform random	$[0, \pi/8]^2$
2-pendulum	$\dot{\theta}$	2	Constant	0
1-pendulum	θ	1	Uniform random	$[0, \pi/2]$
1-pendulum	$\dot{\theta}$	1	Uniform random	[0,0.5]
1&2-pendulum	m	1	Constant	1
1&2-pendulum	g	1	Constant	9.8

Table S4: Initial state of different systems. "Uniform random" means that the variables are sampled with uniform distribution of given range. "Constant" means the variable is initialized as a constant. In the Hénon–Heiles system, after the initialization, the data that do not satisfy the energy $\mathcal{H}(q_x, q_y, p_x, p_y) \in [\frac{1}{12}, \frac{1}{6}]$ will be removed. The value of θ significantly influences the difficulty of solving the pendulum system, for a more detailed discussion, please refer to Fig. S6.

Simulations on Hénon–Heiles system.

Fig. S1 displays more examples of simulations on Hénon–Heiles system, an implement to Fig. 3e in the main text.

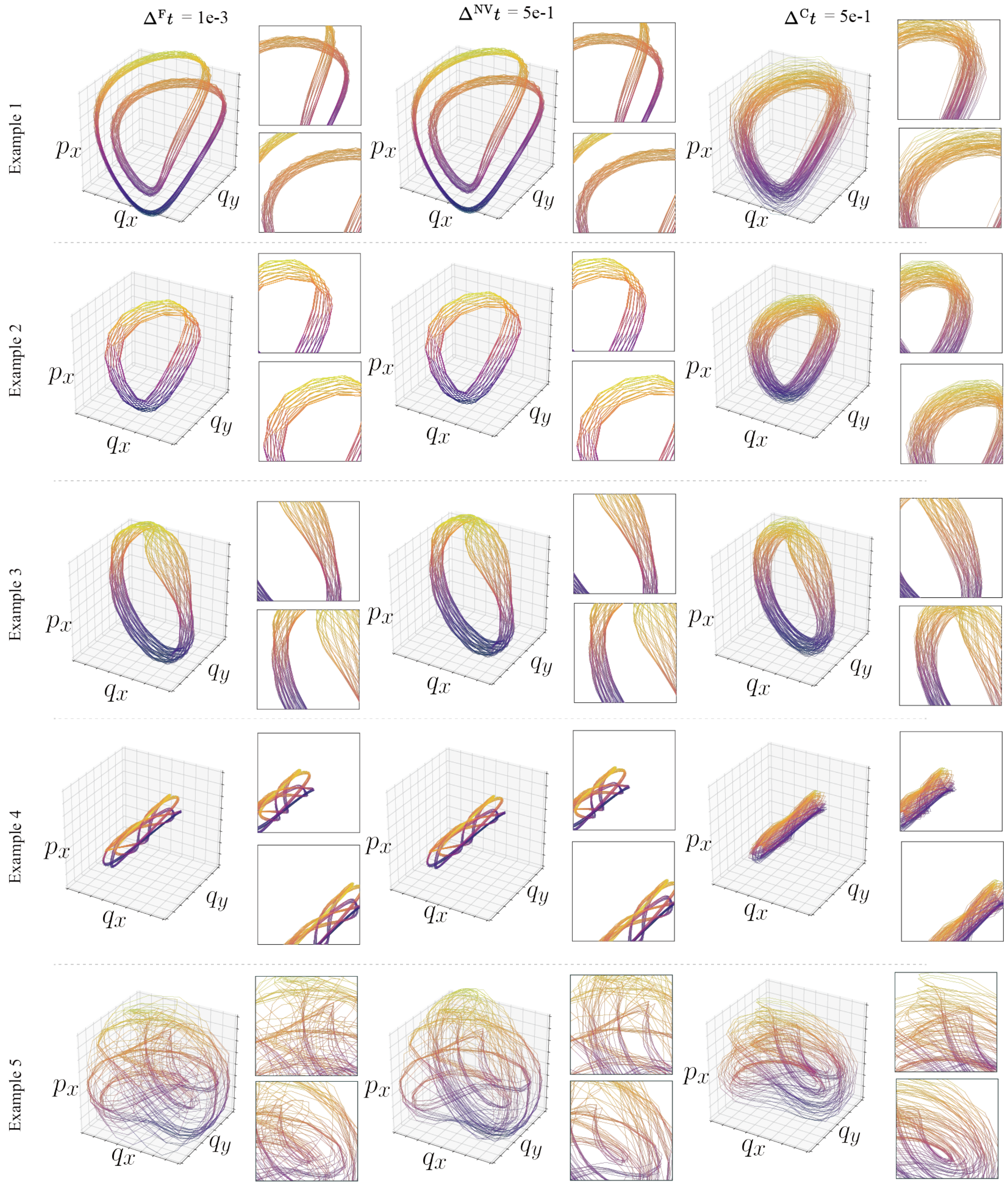

Figure S1: More performance comparison on the Hénon–Heiles system. We provide five additional examples of trajectories projected on the coordinates (q_x, q_y, p_x). NeurVec ($\Delta^{\mathrm{NV}}t = 5e-1$) produces the most orbits similar to $\Delta^{\mathrm{F}}t = 1e-3$.

Time series histogram.

Fig. S2 displays the time series histogram of θ and $\dot{\theta}$ in the elastic pendulum, i.e., Eq. (10) in the main text, and q_x and p_x in the Hénon–Heiles system, i.e., Eq. (8) in the main text. The statistical difference of θ and $\dot{\theta}$ among fine step size, coarse step size and NeurVec with coarse step size is not large. Yet that of q_x and p_x is large.

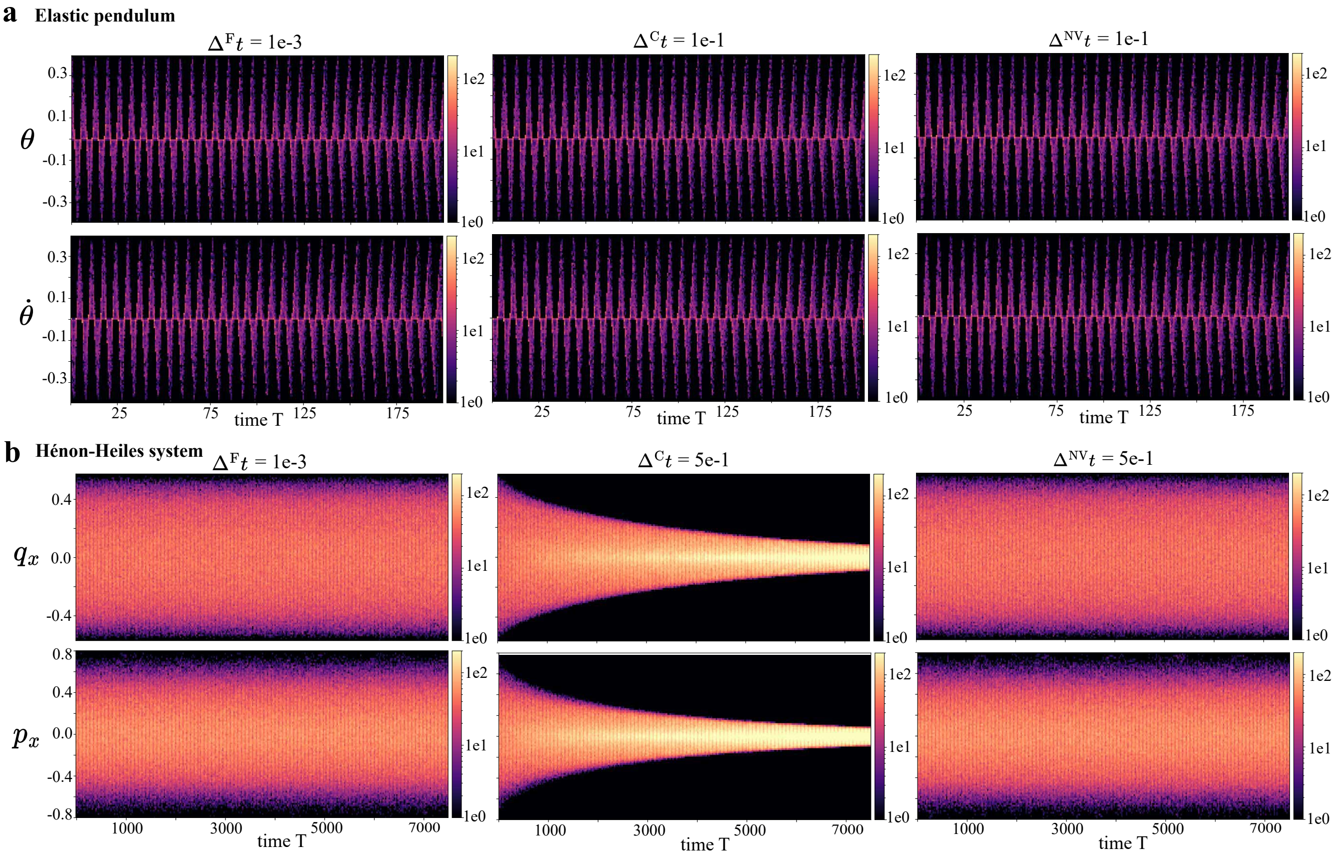

Figure S2: Additional experiments for time series histogram. We visualize the time series histogram of the test set for variables in the elastic pendulum and Hénon–Heiles system. The color represents the number count (the lighter color and the larger frequency). (**a**), θ and $\dot{\theta}$ in the elastic pendulum, i.e., Eq. (10) in the main text. Unlike the results about r and $\dot{r}$ in Fig. 5a of the main text, the θ and $\dot{\theta}$ generated by different step sizes have similar trends, although there are minor differences among them. However, in (**b**) q_x and p_x in the Hénon–Heiles system, i.e., Eq. (8) in the main text, there is a consistent observation for the solutions under different step sizes with Fig. 5b in the main text.

The distribution of training data for 1-link pendulum.

In Fig. S3, we present the training data for the analysis in Figure 6 in the main text, where the background illustrates the difference between the leading error term and NeurVec. The relatively significant differences near the boundary might be attributed to the limited availability of data that encompasses those boundary regions. Furthermore, in regions with limited training data, such as when $\dot{\theta} = -1.0$ and $\theta = 1.5$, the high accuracy demonstrated by our NeurVec also indicates to some extent the generalization capability.

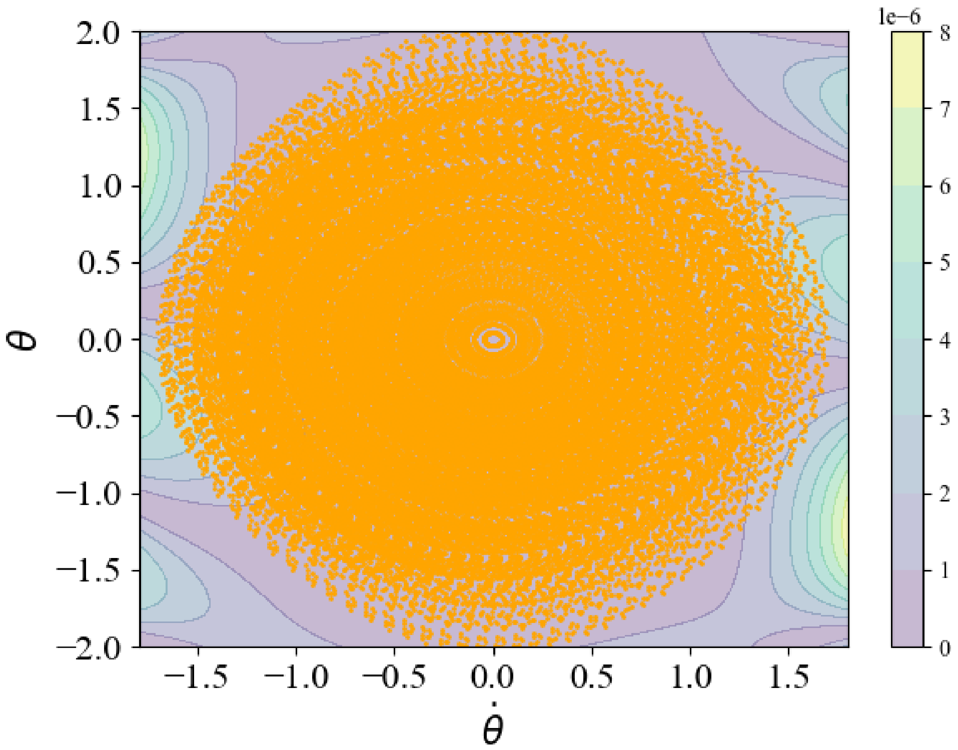

Figure S3: The training data (orange dots) used in 1-link pendulum. The background represents the difference between the leading error term and NeurVec.

Application of NeurVec on the Kuramoto-Sivashinsky equation.

We intend to showcase NeurVec's potential in handling more intricate scenarios by presenting its numerical results on the Kuramoto-Sivashinsky equation (KSE), thus demonstrating its potential to more challenging systems.

We consider KSE on a domain with periodic boundary conditions. The equation is given by:

$$\frac{du}{dt} = -uu_x - u_{xx} - u_{xxxx}, \quad x \in [0, L].$$

Here, L represents the domain length, and we choose $L = \frac{2\pi}{\sqrt{0.085}}$. To solve this equation numerically, we use the exponential time-differencing fourth-order Runge-Kutta method (ETDRK4) as a forward scheme S. The spatial variable x is discretized with a resolution of 48 uniformly spaced points on the interval $[0, L]$.

Our training dataset consists of a single trajectory with an initial state given by $u(x,0) = \cos((\pi/L)x)$. This trajectory is generated with a time step size of $\Delta^{\mathrm{F}} t = 2 \times 10^{-2}$, and the model time reaches $T = 200$k. For testing, we have reference simulations with a smaller time step size of 5×10^{-3}.

NeurVec is trained using the simulations with $\Delta^{\mathrm{F}} t = 2 \times 10^{-2}$ to learn the error correction of ETDRK4 with a coarse time step size $\Delta^{\mathrm{NV}} t = 1$. To assess accuracy, we use the mean square error (MSE) between the reference solution and the simulated solution of $\Delta^{\mathrm{NV}} t = 1$, averaged over 20 different initializations.

Fig. S4a presents the MSE, where the solid curves and the shaded areas represents the mean and one standard deviation, respectively, calculated from 20 simulation runs. We can observe that NeurVec manages to maintain short-term accuracy, closely matching the reference solution, up until the model time reaches $T = 75$. However, beyond this point, NeurVec starts to deviate significantly from the reference solution, yet still maintains smaller error than the simulations of $\Delta^{\mathrm{C}} t = 1$. Fig. S4b shows two examples of the visualized solution for a short period of time. Even though the highly chaotic nature of KSE, NeurVec can still maintain the short-term accuracy as the reference one.

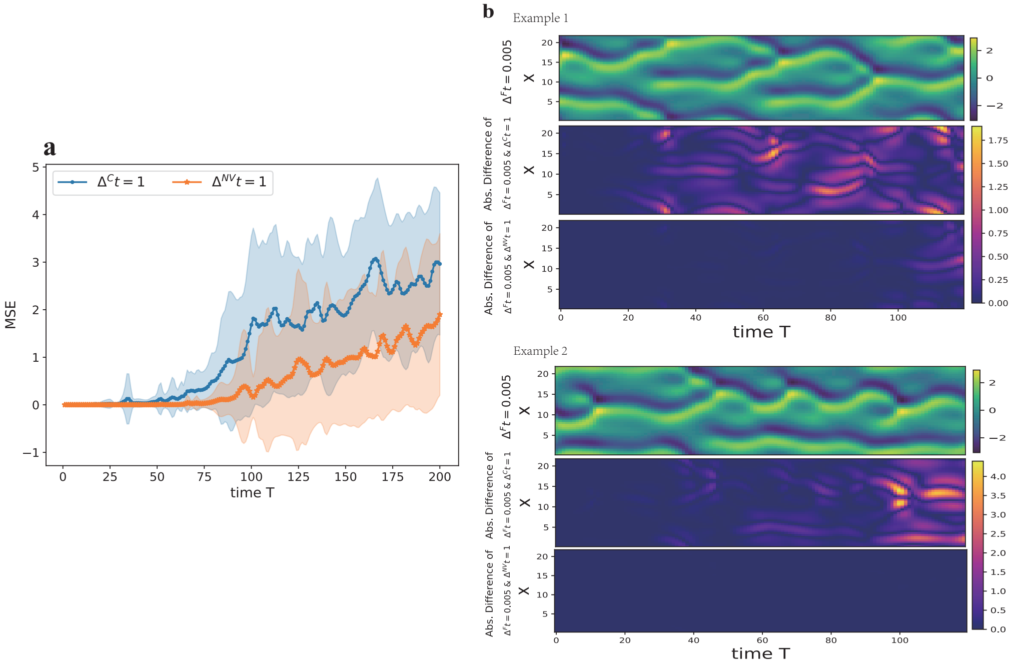

Figure S4: a, The mean square error (MSE) between the reference solution and the numerical solutions with different configurations (step size $\Delta^{\mathrm{C}} t = 1$ and NeurVec with $\Delta^{\mathrm{NV}} t = 1$) for KSE. **b**, we provide two examples of different initializations and plot the absolute differences.

Impact of neural network structures on ϵ.

The ϵ is used to denote the runtime ratio of NeurVec to the scheme S. This section delves into how the neural network structures influence the value of ϵ.

Taking 2-link pendulum system with the Fourth-Order Runge-Kutta method as an example, in Fig. S5, we present the values of ϵ in neural networks under different hidden dimensions, numbers of layers, and activation functions on one A100 GPU. We can observe that the choice of neural network structure significantly impacts the acceleration effect of the neural network on the dynamical system. Specifically,

(1) For the hidden dimension, we use a default setting of 1024. From Fig. S5, we can find that larger hidden dimensions lead to slower inference speed of the neural network.

(2) Regarding the number of layers, we notice that this variable has a greater influence on inference speed compared to the hidden dimension. When the number of layers reaches 4, ϵ is more than twice that of our default setting (with 2 layers). Therefore, selecting the number of layers requires more careful consideration.

(3) As for the activation function, different choices also introduce variations in the model's inference efficiency. Despite the higher inference cost of the "Rational" activation function, it equips the model with strong non-linear fitting capabilities to tackle the complex representation learning of dynamical systems. When the neural networks operate without an activation function (i.e., "Identity"), or when using ReLU, they experience a performance drop of approximately 1∼2 orders of magnitude in terms of MSE on the test set, although they exhibit slightly faster inference speeds.

For the dynamical systems considered in this paper, we find that selecting a hidden dimension of 1024, 2 layers, and the "Rational" activation function provides a sufficiently favorable acceleration effect for solving these systems. When addressing more intricate scenarios in the future, such as complex equations or higher energy levels, careful consideration of the model's structure will be necessary to enable NeurVec to deliver optimal acceleration performance.

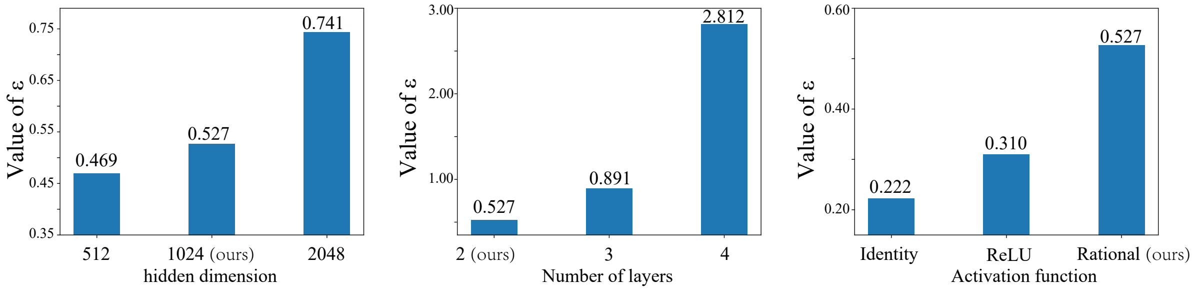

Figure S5: The value ϵ with different network structures.

Exploring NeurVec's performance on trajectories with varying energy levels

In order to explore NeurVec's ability to handle trajectories with varying energy levels, we kept all variables fixed in initial conditions except for θ in Elastic Pendulum and the 2-link Pendulum, and varied the range of θ by changing its sampling interval to investigate the boundaries of NeurVec's capabilities. The sampling interval is $[0, \theta_{\max}]$. Generally, as the upper bound $\theta_{\max}$ of the initial θ increases, trajectories with higher energy levels will be generated, posing a greater challenge for the neural network's learning process. In our paper, $\theta_{\max}$ was set to $\frac{\pi}{8}$. Specifically, for the Elastic Pendulum, θ was uniformly sampled from $[0, \frac{\pi}{8}]$ in the initial conditions. For the 2-link Pendulum, θ was sampled from a two-dimensional uniform distribution $[0, \frac{\pi}{8}]^2$.

Fig. S6 visually represents NeurVec's training Mean Squared Error (MSE) at the 300th epoch for different values of $\theta_{\max}$. As mentioned by the reviewer, trajectories with higher energy levels (i.e., larger $\theta_{\max}$) are more challenging to learn due to their increased chaotic behavior. From Fig. S6, we can observe that currently, NeurVec is capable of tackling $\theta_{\max}$ of approximately less than $\frac{\pi}{4}$.

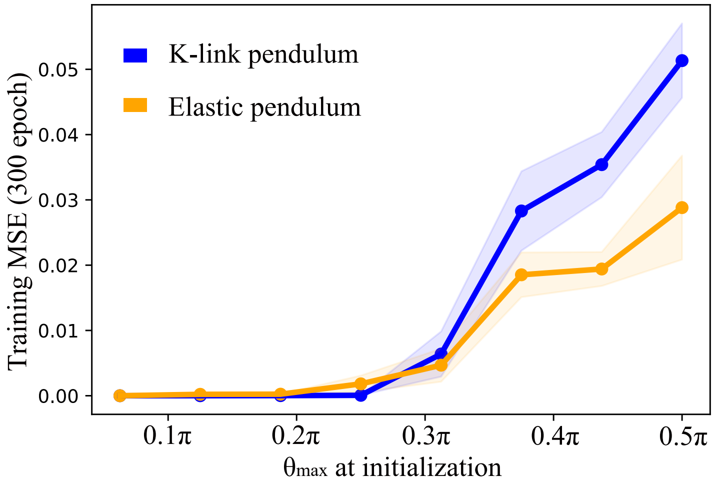

Figure S6: The training MSE on different $\theta_{\max}$.
